# Breast cancer subtype and clinical characteristics in women from Peru

**DOI:** 10.3389/fonc.2023.938042

**Published:** 2023-02-16

**Authors:** Valentina A. Zavala, Sandro Casavilca-Zambrano, Jeannie Navarro-Vásquez, Lizeth I. Tamayo, Carlos A. Castañeda, Guillermo Valencia, Zaida Morante, Mónica Calderón, Julio E. Abugattas, Henry L. Gómez, Hugo A. Fuentes, Ruddy Liendo-Picoaga, Jose M. Cotrina, Silvia P. Neciosup, Katia Roque, Jule Vásquez, Luis Mas, Marco Gálvez-Nino, Laura Fejerman, Tatiana Vidaurre

**Affiliations:** ^1^ Department of Public Health Sciences, University of California, Davis, Davis, CA, United States; ^2^ Instituto Nacional de Enfermedades Neoplásicas, Departamento de Patología, Lima, Peru; ^3^ Instituto Nacional de Enfermedades Neoplásicas, Departamento de Investigación, Lima, Peru; ^4^ Department of Public Health Sciences, The University of Chicago, Chicago, IL, United States; ^5^ Instituto Nacional de Enfermedades Neoplásicas, Departamento de Oncología Médica, Lima, Peru; ^6^ Instituto Nacional de Enfermedades Neoplásicas, Departamento de Cirugía de Mamas y tumores Blandos, Lima, Peru; ^7^ Instituto Nacional de Enfermedades Neoplásicas, Banco de Tumores, Lima, Peru; ^8^ University of California Davis Comprehensive Cancer Center, University of California, Davis, Davis, CA, United States

**Keywords:** breast cancer, genetic ancestry, Hispanics/Latinas, Indigenous American, tumor subtype

## Abstract

**Introduction:**

Breast cancer is a heterogeneous disease, and the distribution of the different subtypes varies by race/ethnic category in the United States and by country. Established breast cancer-associated factors impact subtype-specific risk; however, these included limited or no representation of Latin American diversity. To address this gap in knowledge, we report a description of demographic, reproductive, and lifestyle breast cancer-associated factors by age at diagnosis and disease subtype for The Peruvian Genetics and Genomics of Breast Cancer (PEGEN-BC) study.

**Methods:**

The PEGEN-BC study is a hospital-based breast cancer cohort that includes 1943 patients diagnosed at the Instituto Nacional de Enfermedades Neoplásicas in Lima, Peru. Demographic and reproductive information, as well as lifestyle exposures, were collected with a questionnaire. Clinical data, including tumor Hormone Receptor (HR) status and Human Epidermal Growth Factor Receptor 2 (HER2) status, were abstracted from electronic medical records. Differences in proportions and mean values were tested using Chi-squared and one-way ANOVA tests, respectively. Multinomial logistic regression models were used for multivariate association analyses.

**Results:**

The distribution of subtypes was 52% HR+HER2-, 19% HR+HER2+, 16% HR-HER2-, and 13% HR-HER2+. Indigenous American (IA) genetic ancestry was higher, and height was lower among individuals with the HR-HER2+ subtype (80% IA *vs.* 76% overall, *p*=0.007; 152 cm *vs.* 153 cm overall, *p*=0.032, respectively). In multivariate models, IA ancestry was associated with HR-HER2+ subtype (OR=1.38,95%CI=1.06-1.79, *p*=0.017) and parous women showed increased risk for HR-HER2+ (OR=2.7,95%CI=1.5-4.8, *p*<0.001) and HR-HER2- tumors (OR=2.4,95%CI=1.5-4.0, *p*<0.001) compared to nulliparous women. Multiple patient and tumor characteristics differed by age at diagnosis (<50 vs. >=50), including ancestry, region of residence, family history, height, BMI, breastfeeding, parity, and stage at diagnosis (*p*<0.02 for all variables).

**Discussion:**

The characteristics of the PEGEN-BC study participants do not suggest heterogeneity by tumor subtype except for IA genetic ancestry proportion, which has been previously reported. Differences by age at diagnosis were apparent and concordant with what is known about pre- and post-menopausal-specific disease risk factors. Additional studies in Peru should be developed to further understand the main contributors to the specific age of onset and molecular disease subtypes in this population and develop population-appropriate predictive models for prevention.

## Introduction

Globally, breast cancer is the most commonly diagnosed cancer and the leading cause of cancer death in women (1, 2). Breast cancer risk and mortality vary based on several risk factors. Age, race/ethnicity category, family history, genetics, lifestyle, anthropometric, reproductive, and hormonal factors have been associated with the risk of developing breast cancer (3–5). In addition, tumor subtype, socioeconomic status, education level, and access to care have been shown to impact mortality after diagnosis (6, 7). Analyses stratified by race/ethnicity category have shown that despite sharing risk factors for developing breast cancer, disease risk, clinical characteristics, and risk of mortality differ between populations (6, 8–10). For example, U.S. Hispanics/Latinas (H/Ls) are less likely to develop breast cancer than non-Hispanic White (NHW) and African American women (11). However, after diagnosis, H/L women are at higher risk of mortality compared with NHW women (12).

The use of gene expression profiles for molecular classification of breast cancer tumors (i.e., PAM50) has identified three main intrinsic subtypes: Luminal (A and B), HER2-enriched, and Basal-like (13, 14). A combination of immunohistochemical markers for estrogen receptor (ER), progesterone receptor (PR), and human epidermal growth factor 2 (HER2) are routinely used in clinic to classify tumors into these subtypes and to provide relevant information for individualized therapeutic decision making. Hormone receptor (HR) positive tumors, defined by ER and/or PR expression, are classified as HR+HER2− and HR+HER2+, based on the HER2 expression status, and are overrepresented among luminal intrinsic subtypes. HR−HER2+ and HR−HER2− are overrepresented among HER2-enriched and basal-like subtypes, respectively. Besides chemotherapy, patients with an HR+ disease diagnosis can benefit from endocrine therapy, such as tamoxifen or aromatase inhibitors (15), whereas patients with HER2+ tumors can be treated with anti-HER2 therapy (mainly trastuzumab and pertuzumab) (16). For the HR−HER2− subtype, treatment options are limited. Currently, these patients receive systemic therapy, although targeted therapies, such as PARP and immune checkpoint inhibitors, are being evaluated in clinical trials and approved for BRCA1 and BRCA2 mutation carriers (17).

Multiple studies have suggested heterogeneity in the association between established breast cancer risk factors and tumor subtype. Family history of breast cancer in a first-degree relative is associated with increased breast cancer risk (3, 18, 19), and specific patterns of cancer family history increase the risk of particular tumor subtypes (20, 21). For example, having one first-degree relative with a history of breast cancer was shown to be associated with increased risk of HR+ subtypes, whereas having two or more was associated with increased risk of HR− disease. (20, 21). However, some studies have failed to confirm these findings (3, 22–24). Among reproductive factors, early menarche, and late menopause increase the risk of developing breast cancer (3, 20, 25–27) with no evidence of heterogeneity by tumor subtype (3, 20, 26, 27). Parity is associated with reduced risk of HR+ disease (3, 19, 20, 27–33) and increased odds for developing HR− subtypes (3, 24, 27, 31, 33–35) in populations of European and African origins. Some studies have reported that older age at first full-term pregnancy was associated with increased risk of HR+ disease (27, 28, 30). Longer breastfeeding history is associated with reduced breast cancer risk with lower odds of developing HR− tumors (19, 20, 25–28, 30–34, 36). Among African Americans, prolonged lactation is associated with reduced risk of HR−, but not HR+ disease, with an increased risk of HR− disease among parous women who have not breastfed (34, 37). This observation has also been described among NHW women (32). Reports on lifestyle factors, such as alcohol intake and smoking history, have shown heterogeneity by tumor subtype, with a stronger association with HR+HER2− subtypes (3, 38).

The effects of some of the abovementioned factors are different among pre- and post-menopausal women. Controversial evidence shows that high BMI (obesity) is protective against breast cancer in premenopausal women, and conversely, it suggests that obesity increases the risk in postmenopausal women (39, 40), especially for HR+ subtypes (41–43). Other factors known to affect breast cancer risk in both groups in the same direction can present different magnitudes of the effect by menopausal status, such as alcohol intake (44), physical activity (45, 46), and breastfeeding (47).

Previous studies have assessed the association of breast cancer risk with numerous structural, social, environmental, and genetic factors (4, 48–50); however, these studies are primarily composed of individuals of European origin. Few breast cancer studies describe patient characteristics in Latin America (26, 51–54), a region characterized by cultural and genetic heterogeneity (55–57). For example, Indigenous American genetic ancestry estimates vary across different Latin American countries, ranging between ~5% in Puerto Rico and ~80% in Peru and Bolivia (56–58). Previous studies have identified that the degree of Indigenous American genetic ancestry may modify the magnitude and direction of association with currently known breast cancer risk variants among H/L women (59) and is associated with differential lifestyle risk factors (60). Latin American cohorts with high proportions of Indigenous American ancestry are underrepresented in breast cancer research (61).

The Peruvian Genetics and Genomics of Breast Cancer Study (PEGEN-BC) is a hospital-based cohort including patients from the Instituto Nacional de Enfermedades Neoplásicas (INEN) in Lima, Peru. We have previously described the distribution of demographic, anthropometric, reproductive, lifestyle, and clinical factors for 1,312 breast cancer participants, with an emphasis on the distribution by breast tumor subtypes (62). Moreover, we reported that increasing Indigenous American ancestry is associated with higher odds of developing the HR−HER2+ subtype (62). The current report aims to provide a more complete and updated description of these variables by tumor subtype and age at diagnosis, including a total of 1,943 breast cancer patients, highlighting potential heterogeneity in the latter categories.

## Methods

### Study participants

The Peruvian Genetics and Genomics of Breast Cancer Study (PEGEN-BC) is a hospital-based cohort study. As of April 2022, we have recruited 1,943 participants from the INEN in Lima, Peru. Women were invited to participate if they had a diagnosis of invasive breast cancer in 2010 or later and were between 21 and 79 years of age when diagnosed. A blood sample was drawn by a certified phlebotomist at the INEN central laboratory. The present report includes analyses with a subset of 1,796 patients with available genetic ancestry estimates (63). This study was approved by the INEN and the University of California Davis Institutional Review Boards. All individuals provided written informed consent to participate.

### Data collection

Each PEGEN-BC participant completed a standardized survey administered by a trained research coordinator at INEN. The survey includes questions regarding anthropometric (weight and height), demographic (place of birth and residence), lifestyle (alcohol intake and smoking history), and reproductive (menopause status, age at first pregnancy, number of full-term pregnancies, and breastfeeding history) variables, and family history of breast cancer. Weight and height were assessed by trained nurses/professionals at INEN at the time of diagnosis. Body mass index (BMI) was calculated as weight (kilograms) divided by height (meters) squared and categorized as underweight (BMI < 18.5 kg/m ^2^), normal (BMI ≥ 18.5 < 25 kg/m^2^), overweight (BMI ≥ 25 < 30 kg/m ^2^), and obese (BMI ≥ 30 kg/m^2^). Alcohol use was assessed as the self-reported frequency of glasses of alcohol consumed per day and categorized as < 1 glass/day, > 1 glass/day, and non-drinker (never). Smoking status was classified into “ever” (current and former) and “never.” If there was a history of familial breast cancer, the relative (i.e., mother, sister, and aunt) was indicated to determine cases with breast cancer family history in a first-degree relative. Clinical variables, including ER, PR, HER2, lymph node status, tumor grade, and clinical stage, were extracted from electronic records.

Genetic ancestry estimates for 1,796 PEGEN-BC participants were available from a previous study (63). Briefly, genome-wide genotype data obtained with the Affymetrix Precision Medicine Array were pruned using PLINK v.1.9 (64) [window size = 50, number of variants = 5, variance inflation factor threshold = 2] and merged with data from four reference populations from the 1000 Genomes project (65): Admixed Americans (Peru, Colombia, Mexico, Puerto Rico), Europeans (Americans with Northern and Western European Ancestry, Italy, Spain, Finland, Scotland), East Asians (China, Japan, Vietnam), and African populations (Nigeria, Kenya, Gambia, Sierra Leone). Individual continental, global genetic ancestry was estimated using ADMIXTURE (66) (unsupervised, *k* = 4), including 122,605 independent variants. The PEGEN-BC study includes a large proportion of patients with > 98% Indigenous American ancestry, as previously reported (62), and therefore provides a source of non-admixed reference samples for this component.

Tumoral tissues were obtained from core biopsy or freshly resected invasive breast cancers pre-treatment that were formalin-fixed and paraffin-embedded following standard protocols at INEN. Tumor subtypes were defined using immunohistochemistry (IHC) markers by a certified pathologist at INEN. HR positivity was defined at 1% or more cells showing ER and/or PR staining. HER2 positivity was defined as 3+ staining by IHC or by gene amplification detected by fluorescence *in situ* hybridization following a 2+ (borderline) IHC result. These markers were used to classify tumors as HR+HER2−, HR+HER2+, HR−HER2+, and HR−HER2−. Two independent pathologists from the University of California San Francisco reviewed the IHC slides at INEN for a subset of 52 patients. The concordance rate was 100% for ER, 87% for PR, and 85% for HER2. Most of the discordant calls for HER2 were scored as “negative” or 1+ at INEN and 2+ by the independent pathologists. Immunohistochemical subtype classification was not available for 141 samples (7%).

### Statistical analysis

We performed descriptive analyses of available demographic, anthropometric, reproductive, and clinical characteristics by breast cancer subtype. Differences in characteristics between tumor subtypes were tested by means of one-way ANOVA for normally distributed continuous variables and Chi-squared tests for categorical variables. Age at first full-term pregnancy presented a non-normal distribution; therefore, it was log_2_ transformed. The correlation between genetic ancestry and continuous and categorical variables was performed using Pearson’s correlation coefficient test and Point-Biserial Correlation Coefficient, respectively. Multinomial logistic regression models were used to calculate odds ratios (ORs) and 95% confidence intervals (CI) for the association of multiple variables and subtype-specific breast cancer. East Asian and African ancestry proportions were not included in multivariable models due to the low contribution of these components and high correlation with the Indigenous American/European axis of ancestry variation. *P*-values (*P*) <= 0.05 were considered statistically significant. All analyses were conducted in R v.3.6.0 (67).

## Results

### Demographics, anthropometrics, and lifestyle factors in the PEGEN-BC study by tumor subtype

**Figure 1:**
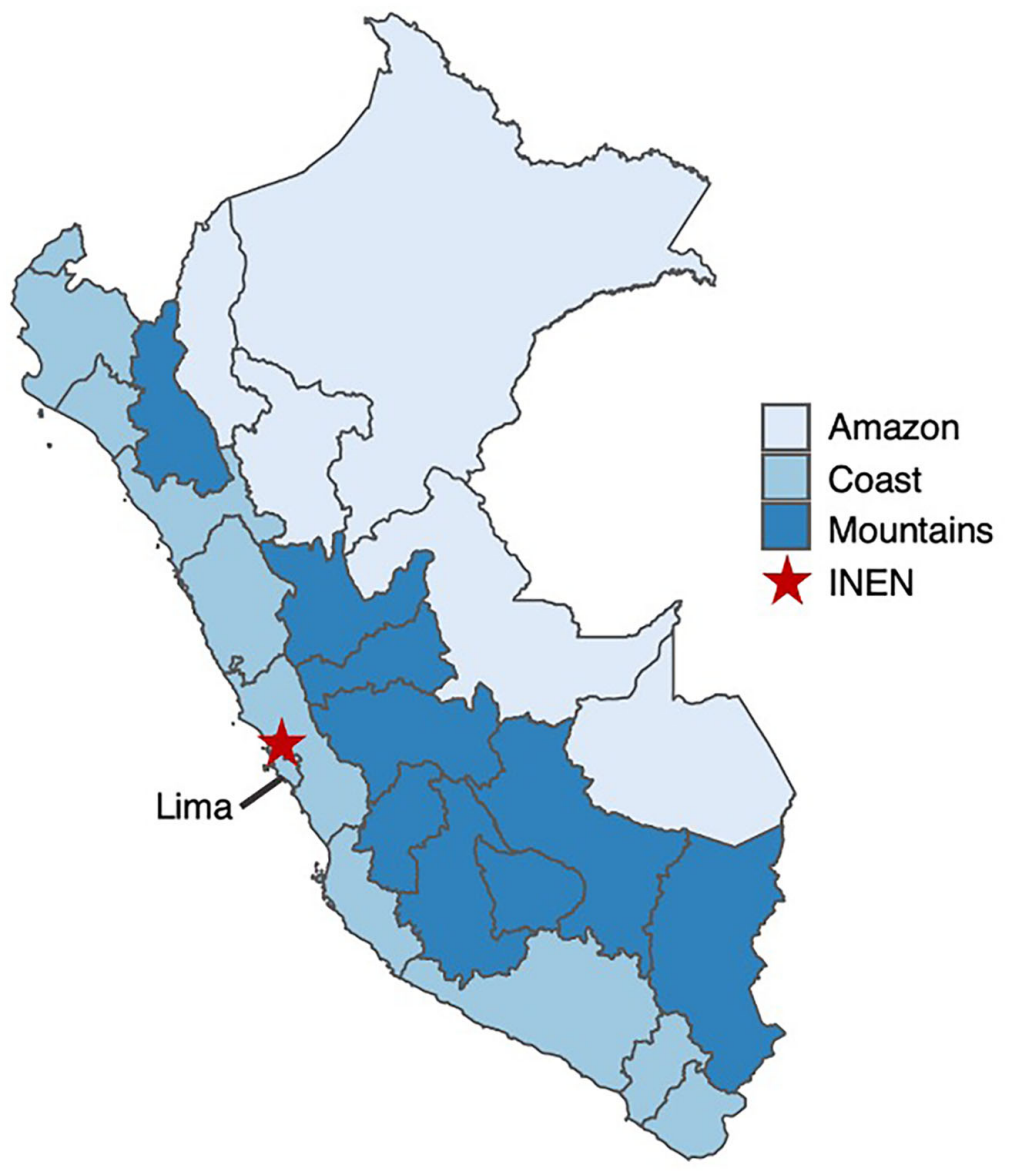
Biogeographical regions of Peru. Red star shows the location of INEN. This figure was created using the ggmap, maps, and mapdata R packages.

The most common breast cancer subtype among PEGEN-BC study participants was HR+HER2− (52.4%), followed by HR+HER2+ (18.7%), HR−HER2− (16.0%), and HR−HER2+ (12.9%) (**Table 1**). The average age at diagnosis was 49.8 years (*SD* = 11), and differences by tumor subtype were not statistically significant (*p* = 0.087). PEGEN-BC study patients included individuals born in the three main biogeographic regions of Peru (**Figure 1**): The Coastal (55.5%), Mountainous (36.4%), and Amazonian (7.5%) regions. Less than 1% of the patients were born in another country (mainly Venezuela). These groups did not show statistically significant differences in their distribution by tumor subtype (**Table 1**). Most patients resided in the Coastal region (7%), and differences in the proportion of patients who resided in each biogeographic area by tumor subtype category were not statistically significant (**Table 1**).

**Table 1:** Distribution of demographic, lifestyle, and anthropometric characteristics of PEGEN-BC patients overall and by tumor subtype.

Variable	Overall	HR+HER2−	HR+HER2+	HR−HER2+	HR−HER2−	*p*-value
Number of patients, *N* (%)	1943 (100)*	945 (52.4)	337 (18.7)	232 (12.9)	288 (16.0)	
Demographic variables						
Age at diagnosis in years, mean (SD)	49.8 (11.0)	50.3 (11.1)	48.9 (10.2)	50.0 (10.9)	48.8 (12.0)	0.087
Missing, *N* (%)	7 (0.4)	1 (0.1)	0 (0.0)	0 (0.0)	1 (0.34)	
Percent genetic ancestry**, mean (SD)						
Indigenous American	76.5 (16.9)	75.3 (17.4)	76.6 (16.8)	79.5 (14.78)	77.6 (16.5)	0.007
European	18.0 (12.5)	18.7 (12.9)	17.8 (12.0)	16.2 (11.36)	17.4 (12.5)	0.036
African	4.2 (7.7)	4.1 (7.6)	4.6 (8.9)	3.6 (6.26)	4.1 (7.1)	0.494
East Asian	1.4 (6.6)	1.9 (8.6)	1.0 (3.7)	0.8 (2.42)	0.9 (3.1)	0.026
Missing, *N* (%)	147 (7.6)	47 (5.0)	22 (6.5)	10 (4.3)	21 (7.3)	
Region of birth, *N* (%)						
Amazonian	145 (7.5)	69 (7.3)	22 (6.5)	18 (7.8)	23 (8.0)	0.737
Coastal	1078 (55.5)	522 (55.2)	178 (52.8)	124 (53.4)	165 (57.3)	
Mountains	708 (36.4)	346 (36.6)	137 (40.7)	88 (37.9)	98 (34.0)	
Other country***	12 (0.6)	8 (0.8)	0 (0.0)	2 (0.9)	2 (0.7)	
Missing	0 (0.0)	0 (0.0)	0 (0.0)	0 (0.0)	0 (0.0)	
Region of residence, *N* (%)						
Amazonian	120 (6.2)	56 (5.9)	18 (5.3)	11 (4.7)	24 (8.3)	0.138
Coastal	1530 (78.7)	757 (80.1)	264 (78.3)	174 (75.0)	216 (75.0)	
Mountains	293 (15.1)	132 (14.0)	55 (16.3)	47 (20.3)	48 (16.7)	
Missing	0 (0.0)	0 (0.0)	0 (0.0)	0 (0.0)	0 (0.0)	
Anthropometric and lifestyle variables						
Weight in kg, mean (SD)	64.8 (12.3)	65.2 (12.4)	64.8 (11.9)	63.6 (11.6)	64.6 (12.7)	0.350
Missing, *N* (%)	41 (2.1)	15 (1.6)	4 (1.2)	7 (3.0)	7 (2.4)	
Height in m, mean (SD)	153.3 (6.6)	153.3 (6.5)	153.7 (6.4)	152.1 (6.5)	153.4 (6.5)	0.032
Missing, *N* (%)	47 (2.4)	17 (1.8)	7 (2.1)	10 (4.3)	6 (2.1)	
BMI in kg/m^2^, mean (SD)	27.54 (4.8)	27.7 (4.8)	27.4 (4.7)	27.5 (4.9)	27.4 (4.8)	0.705
Missing, *N* (%)	56 (2.9)	22 (2.3)	7 (2.1)	10 (4.3)	8 (2.8)	
BMI categorized, *N* (%)						
Underweight***	22 (1.1)	11 (1.2)	4 (1.2)	4 (1.7)	2 (0.7)	0.852
Normal	564 (29.0)	263 (27.8)	109 (32.3)	65 (28.0)	84 (29.2)	
Overweight	779 (40.1)	383 (40.5)	129 (38.3)	93 (40.1)	117 (40.6)	
Obese	522 (26.9)	266 (28.1)	88 (26.1)	60 (25.9)	77 (26.7)	
Alcohol intake, *N* (%)						
< 1 glass/day	1335 (68.7)	655 (69.3)	223 (66.2)	159 (68.5)	186 (64.6)	0.603
> 1 glass/day	144 (7.4)	66 (7.0)	25 (7.4)	19 (8.2)	26 (9.0)	
Never	446 (23.0)	213 (22.5)	88 (26.1)	51 (22.0)	75 (26.0)	
Missing	18 (0.9)	11 (1.2)	1 (0.3)	3 (1.3)	1 (0.3)	
Smoking history, *N* (%)						
Never	1382 (71.1)	655 (69.3)	242 (71.8)	179 (77.2)	212 (73.6)	0.087
Ever	543 (27.9)	280 (29.6)	94 (27.9)	50 (21.6)	75 (26.0)	
Missing	18 (0.9)	10 (1.1)	1 (0.3)	3 (1.3)	1 (0.3)	

*Immunohistochemical subtype classification was not available for 141 samples (7%). **Estimates of individual continental ancestry were unavailable for 147 patients (7.6%). ***Category not included in the Chi-square test due to small sample size. “Missing” categories were excluded from tests.

**Figure 2:**
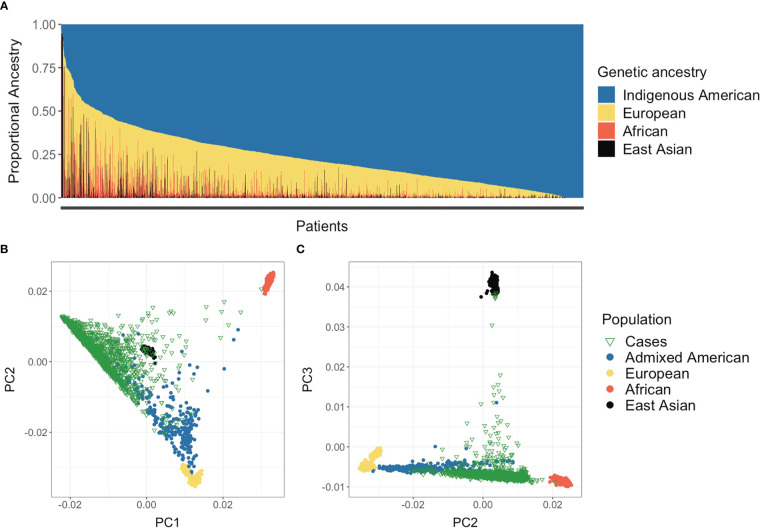
Population genetic structure of the PEGEN-BC study participants. **(A)** ADMIXTURE continental ancestry estimates obtained in unsupervised analysis, assuming *K* = 4. **(B, C)** Principal components analysis (PCA) including breast cancer patients and 1000 Genomes Project individuals. The first three principal components are shown.

Estimates of individual continental genetic ancestry were available for 1,796 patients. Average Indigenous American ancestry among patients was 76.5%, followed by 18.0% European, 4.2% African, and 1.4% East Asian (**Table 1**). Furthermore, 92% of PEGEN-BC study participants had > 50% of Indigenous American ancestry, 25% at least 90%, and 12% at least 95% of Indigenous American ancestry (**Figure 2A**). Seven patients (0.4%) had more than 50% of East Asian ancestry, and eight (0.4%) had more than 50% African ancestry. Principal components analysis showed that the PEGEN-BC patients defined the Indigenous American cluster along principal component (PC) 1 when compared against 1000 Genomes Project reference populations (**Figures 2B, C**), reflecting the high degree of Indigenous American genetic ancestry that characterizes this cohort.

We found that the average Indigenous American ancestry proportion of participants was different across tumor subtypes. Individuals diagnosed with HR−HER2+ tumors showed the highest average proportion of Indigenous American ancestry (79.5%, *SD* = 15) (**Table 1**).

The average height of patients was 153.3 cm (*SD* = 6.6), with lower average height among patients diagnosed with HR−HER2+ tumors compared with all other subtypes (152.1 *vs.* ~153.6 cm, *p* = 0.032). There were no statistically significant differences in weight or BMI by tumor subtype, with a large overall proportion of patients being overweight (40.1%) (**Table 1**).

Most PEGEN-BC patients (68.7%) reported low levels of alcohol consumption (< 1 glass/day), whereas 7.4% reported consuming more than one glass per day. Moreover, 27.9% of participants reported being a current or past smoker. There was no statistically significant association between alcohol consumption, smoking history, and tumor subtype (**Table 1**).

Demographic, anthropometric, and lifestyle variables that did not show statistically significant differences by tumor subtypes did not show significant differences by HR status either (**Supplementary Table S1**).

### Reproductive variables by tumor subtype

The average age at menarche among PEGEN-BC patients was 12.9 years (*SD* = 1.7), the average age at first full-term pregnancy was 23.2 years (*SD* = 5.7), and the average number of full-term pregnancies was 2.42 (*SD* = 1.8). Most study participants reported having had at least one child (83.5%), and 80% of parous women had at least two children (**Table 2**). The frequency of parous women and number of births differed by tumor subtype, being higher among HR− tumors (*p* < 0.001) (**Table 2**).

**Table 2:** Distribution of reproductive variables overall and by tumor subtype.

Variable	Overall	HR+HER2−	HR+HER2+	HR-HER2+	HR−HER2−	*p*-value
Number of patients, *N* (%)	1943 (100)	945 (52.4)	337 (18.7)	232 (12.9)	288 (16.0)	
Age at menarche in years, mean (SD)	12.9 (1.7)	12.9 (1.8)	12.9 (1.7)	13.1 (1.7)	13.0 (1.7)	0.364
Missing, *N* (%)	34 (1.8)	17 (1.8)	3 (0.9)	7 (3.0)	2 (0.7)	
Parous, yes, *N* (%)	1623 (83.5)	765 (81.0)	273 (81.0)	207 (89.2)	263 (91.3)	< 0.001
Missing, *N* (%)	63 (3.2)	32 (3.4)	10 (3.0)	9 (3.9)	4 (1.4)	
Age at first full-term pregnancy in years, mean (SD)	23.2 (5.7)	23.5 (5.8)	23.0 (5.3)	22.9 (6.1)	22.5 (5.4)	0.095
Missing*, *N* (%)	72 (4.4)	40 (5.2)	7 (2.6)	13 (6.3)	6 (2.3)	
Parity, mean (SD)	2.4 (1.8)	2.3 (1.8)	2.3 (1.9)	2.7 (1.8)	2.7 (1.7)	0.002
Missing*, *N* (%)	6 (0.4)	4 (0.5)	1 (0.4)	1 (0.5)	0 (0.0)	
Parity categories, *N* (%)						
No children	275 (14.2)	156 (16.5)	57 (16.9)	19 (8.2)	22 (7.6)	< 0.001
1 child	316 (16.3)	162 (17.1)	47 (13.9)	38 (16.4)	46 (16.0)	
2–3 children	888 (45.7)	410 (43.4)	161 (47.8)	105 (45.3)	148 (51.4)	
>3 children	413 (21.3)	189 (20.0)	64 (19.0)	63 (27.2)	69 (24.0)	
Missing, *N* (%)	51 (2.6)	28 (3.0)	8 (2.4)	7 (3.0)	3 (1.0)	
Breastfed*, yes, *N* (%)	1563 (96.3)	736 (96.2)	264 (96.7)	200 (96.6)	255 (97.0)	0.967
Missing*, *N* (%)	2 (0.1)	1 (0.1)	0 (0.0)	0 (0.0)	0 (0.0)	
Postmenopausal, *N* (%)	1681 (86.5)	839 (88.8)	287 (85.2)	198 (85.3)	240 (83.3)	0.016
Missing, *N* (%)	23 (1.2)	12 (1.3)	1 (0.3)	5 (2.2)	1 (0.3)	

*Proportion in relation to the total number of parous women. Missing categories were not included in the analysis.

Breastfeeding was a common practice among parous women (96.3%), and we did not observe the differences in the proportion of women who breastfed their children by tumor subtype category (**Table 2**).

More than 85% of women reported being menopausal at recruitment. Patients with HR+HER2− tumors were more likely to report being menopausal than patients with other tumor subtypes (*p* = 0.016). However, since many of these patients had induced menopause due to treatment, we did not consider this variable in subsequent multivariate analyses and stratified by age at diagnosis instead.

All these variables remained significant in analyses stratified by HR status (**Supplementary Table S2**). In addition, age at first full-term pregnancy showed a higher average age among patients diagnosed with HR+ disease compared with HR− (23.4 *vs.* 22.7, *p* = 0.043, **Supplementary Table S2**).

### Clinical characteristics by tumor subtype

Overall, approximately 8% of PEGEN-BC study patients reported a family history of breast cancer in a first-degree relative (**Table 3**). Differences in breast cancer family history by breast cancer subtype were not statistically significant.

**Table 3:** Distribution of clinical characteristics of PEGEN-BC study participants overall and by tumor subtype.

Variable	Overall	HR+HER2−	HR+HER2+	HR−HER2+	HR−HER2−	*p*-value
Number of patients, *N* (%)	1943 (100)*	945 (52.4)	337 (18.7)	232 (12.9)	288 (16.0)	
Positive family history of breast cancer**, *N* (%)	149 (7.7)	84 (8.9)	25 (7.4)	9 (3.9)	23 (8.0)	0.091
Missing	61 (3.1)	19 (2.0)	5 (1.5)	7 (3.0)	2 (0.7)	
Grade, *N* (%)						
1	72 (3.7)	58 (6.1)	6 (1.8)	0 (0.0)	4 (1.4)	< 0.001
2	803 (41.3)	550 (58.2)	117 (34.7)	36 (15.5)	37 (12.8)	
3	1005 (51.7)	317 (33.5)	209 (62.0)	192 (82.8)	239 (83.0)	
Missing	63 (3.2)	20 (2.1)	5 (1.5)	4 (1.7)	8 (2.8)	
Stage, *N* (%)						
I	122 (6.3)	67 (7.1)	18 (5.3)	7 (3.0)	23 (8.0)	< 0.001
II	840 (43.2)	480 (50.8)	134 (39.8)	70 (30.2)	109 (37.8)	
III	798 (41.1)	332 (35.1)	158 (46.9)	137 (59.1)	139 (48.3)	
IV	105 (5.4)	49 (5.2)	19 (5.6)	16 (6.9)	12 (4.2)	
Missing	78 (4.0)	17 (1.8)	8 (2.4)	2 (0.9)	5 (1.7)	
Positive lymph node status, *N* (%)	1249 (64.3)	585 (61.9)	227 (67.4)	176 (75.9)	177 (61.5)	0.002
Missing	90 (4.6)	43 (4.6)	9 (2.7)	7 (3.0)	21 (7.3)	

*Immunohistochemical subtype classification was not available for 141 samples (7%). **In a first-degree relative.

More than 90% of patients were diagnosed with Grades 2 and 3 tumors (**Table 3**). Patients with HR+HER2− tumors were more likely to be diagnosed with Grades 1 and 2 disease, whereas those with HR−HER2+ and HR−HER2− tumors were more likely to be high grade (**Table 3**). Most PEGEN-BC participants were diagnosed with stage II or III disease, with a larger number of stage I and II diagnoses among HR+HER2− patients than those with other subtypes (**Table 3**). Concordant with the distribution of tumor stage, we observed a high proportion of positive lymph node status among patients overall (64.3%), with a statistically significantly higher proportion of lymph node positivity among patients with HR−HER2+ tumors compared with those with other disease subtypes (78.2% *vs.* ~67%) (**Table 3**). Distribution of these variables by HR status is shown in **Supplementary Table S2**.

### Distribution of patient characteristics by age at diagnosis

We compared the distribution of anthropometric, demographic, reproductive, clinical, and lifestyle risk variables between patients diagnosed before the age of 50 years (*N* = 981) and at 50 years or older (*N* = 955). Compared with patients diagnosed at 50 years or older, younger patients had higher average Indigenous American ancestry (78.6 *vs.* 74.3, *p* < 0.001); they were more likely to reside in the Mountainous region (17.3% *vs.* 12.8%, *p* = 0.015), and they were 1.4 cm taller (*p* < 0.001) and had lower prevalence of obesity (25.4% *vs.* 30.0%, *p* = 0.036) (**Table 4**). Additionally, there was a higher proportion of older patients with more than three children compared with the younger group (31% *vs.* 13%, *p* < 0.001), and a larger proportion of younger patients reported breastfeeding their children (98% *vs.* 95%, *p* = 0.001) (**Table 5**). Regarding clinical characteristics, younger patients reported lower family history of breast cancer in a first-degree relative (6.5% *vs.* 9.5%, *p* = 0.02) and presented with more advanced disease (44% diagnosed at stage III compared with 42%, *p* = 0.017) (**Table 5**). We did not observe statistically significant differences in subtype distribution between both age categories.

**Table 4:** Distribution of demographic and anthropometric variables by age at diagnosis categories.

	Age at diagnosis		
Variable	< 50 years old	>= 50 years old	*p*-value
Number of patients, *N* (%)	981 (50.5)	955 (49.2)	
**Demographic variables**			
Age at diagnosis in years, mean (SD)	41.0 (5.9)	58.8 (7.0)	–
Missing, *N* (%)	0 (%)	0 (%)	
Percent genetic ancestry*, mean (SD)			
Indigenous American	78.6 (15.1)	74.3 (18.3)	< 0.001
European	16.84 (11.5)	19.1 (13.3)	< 0.001
African	3.6 (6.6)	4.7 (8.6)	0.004
East Asian	1.0 (4.2)	1.9 (8.4)	0.003
Missing, *N* (%)	72 (7.3%)	73 (7.6%)	
Region of birth, *N* (%)			
Amazonian	71 (7.2)	73 (7.6)	0.904
Coastal	548 (55.9)	526 (55.1)	
Mountains	355 (36.2)	351 (36.8)	
Other country**	7 (0.7)	5 (0.5)	
Missing, *N* (%)	0 (%)	0 (%)	
Region of residence, *N* (%)			
Amazonian	63 (6.4)	57 (6.0)	0.015
Coastal	748 (76.2)	776 (81.3)	
Mountains	170 (17.3)	122 (12.8)	
Missing, *N* (%)	0 (%)	0 (%)	
**Anthropometric and lifestyle variables**			
Weight in kg, mean (SD)	64.8 (12.4)	64.8 (12.3)	0.983
Missing, *N* (%)	17 (1.7)	24 (2.5)	
Height in cm, mean (SD)	154.0 (6.3)	152.6 (6.7)	< 0.001
Missing, *N* (%)	18 (1.8)	29 (3.0)	
BMI in kg/m^2^, mean (SD)	27.3 (4.6)	27.8 (4.9)	0.009
Missing, *N* (%)	24 (2.4)	32 (3.3)	
BMI categorized, *N* (%)			
Underweight ***	6 (0.6)	16 (1.7)	0.036
Normal	305 (31.9)	255 (27.6)	
Overweight	403 (42.1)	375 (40.6)	
Obese	243 (25.4)	277 (30.0)	
Alcohol intake, *N* (%)			
< 1 glass/day	664 (67.7)	665 (69.6)	0.161
> 1 glass/day	84 (8.6)	60 (6.3)	
Never	225 (22.9)	220 (23.0)	
Missing, *N* (%)	8 (0.8)	10 (1.0)	
Smoking history, *N* (%)			
Never	704 (71.8)	674 (70.6)	0.705
Ever	270 (27.5)	270 (28.3)	
Missing, *N* (%)	7 (0.7)	11 (1.2)	

*Estimates of individual continental ancestry were available for 92.6% of patients diagnosed before 50 and 92.3% for patients diagnosed at 50 or above. **Category not included in the Chi-square test due to small sample size.

**Table 5:** Distribution of reproductive and clinical variables by age at diagnosis categories.

	Age at diagnosis		
Variable	< 50 years old	>= 50 years old	*p*-value
Number of patients, *N* (%)	981 (50.5)	955 (49.2)	
Reproductive variables			
Age at menarche in years, mean (SD)	12.9 (1.7)	13.0 (1.7)	0.849
Missing, *N* (%)	15 (1.5)	19 (2.0)	
Parous, yes, *N* (%)	815 (85.9)	802 (86.7)	0.652
Missing, *N* (%)	32 (3.2)	30 (3.1)	
Age at first full-term pregnancy in years, mean (SD)	23.19 (5.53)	23.10 (5.83)	0.747
Missing*, *N* (%)	39 (4.8)	33 (4.1)	
Parity, mean (SD)	2.0 (1.34)	2.8 (2.11)	< 0.001
Missing*, *N* (%)	4 (0.4)	2 (0.2)	
Parity categories, *N* (%)			
No children	146 (14.9)	128 (13.4)	< 0.001
1 child	190 (19.4)	125 (13.1)	
2–3 children	499 (50.9)	385 (40.3)	
> 3 children	122 (12.4)	290 (30.4)	
Missing, *N* (%)	24 (2.4)	27 (2.8)	
Breastfed*, yes, *N* (%)	798 (98.0)	759 (94.6)	0.001
Missing*, *N* (%)	1 (0.1)	1 (0.1)	
Clinical characteristics			
Positive family history of breast cancer**, *N* (%)	62 (6.3)	87 (9.1)	0.020
Missing, *N* (%)	23 (2.3)	38 (4.0)	
Tumor grade, *N* (%)			
1	37 (3.8)	35 (3.7)	0.421
2	393 (40.1)	410 (42.9)	
3	523 (53.3)	482 (50.5)	
Missing, *N* (%)	28 (2.9)	28 (2.9)	
Positive lymph node status, *N* (%)	636 (64.8)	610 (63.9)	0.460
Missing, *N* (%)	49 (5.0)	38 (4.0)	
Stage, *N* (%)			
I	45 (4.6)	77 (8.1)	0.017
II	428 (43.6)	410 (42.9)	
III	417 (42.5)	381 (39.9)	
IV	53 (5.4)	50 (5.2)	
Missing, *N* (%)	38 (3.9)	37 (3.9)	
Tumor subtype, *N* (%)			
HR+HER2−	476 (48.5)	468 (49.0)	0.328
HR+HER2+	178 (18.1)	159 (16.6)	
HR−HER2+	108 (11.0)	124 (13.0)	
HR−HER2−	155 (15.8)	132 (13.8)	
Missing, *N* (%)	64 (6.5)	72 (7.5)	

*Proportion in relation to the total number of parous women. **In a first-degree relative.

Additional stratified analyses comparing demographic, anthropometric, reproductive, and clinical factors by tumor subtype in the two different age groups are included as Supplementary Materials (**Supplementary Tables S3 and S4**). As additional stratification reduced the number of observations per category, we suggest taking these results with caution.

### Correlation between Indigenous American genetic ancestry and other patient and tumor characteristics

We assessed the correlation between Indigenous American ancestry and patient and tumor characteristics to better understand the observed patterns in ancestry distribution and those factors by tumor subtype in the PEGEN-BC study. We observed an inverse correlation between Indigenous American ancestry and age at diagnosis (*r* = −0.15, *p* < 0.001), weight (*r* = −0.11, *p* < 0.001), height (*r* = −0.25, *p* < 0.001), age at first full-term pregnancy (*r* = −0.08, *p* = 0.002), family history of breast cancer in a first-degree relative (*r* = −0.12, *p* < 0.001), smoking history (*r* = −0.11, *p* < 0.001), HR+ status (*r* = −0.06, *p* = 0.012) and a positive correlation with age at menarche (*r* = 0.06, *p* = 0.017) and HER2+ status (*r* = 0.053, *p* = 0.029).

### Multivariable analyses testing the association between demographic, lifestyle factors, and breast cancer subtype

Variables that showed statistically significant associations at the 10% level with tumor subtype in the univariate analyses (**Tables 1**–**3**) were included in a multivariate model, using HR+HER2− as reference (**Table 6**). Indigenous American ancestry remained associated with HR−HER2+ subtype (OR per 25% increment in ancestry = 1.38, 95% CI = 1.06–1.79, *p* = 0.02). Smoking history and height were no longer statistically significantly associated with subtype. Parous women were more likely to be diagnosed with HR−HER2+ (OR = 2.72, 95% CI = 1.53–4.83, *p* < 0.001) and HR-HER2- (OR = 2.47, 95% CI = 1.51–4.04, *p* < 0.001) disease compared with the HR+HER2− subtype. Family history of breast cancer in a first-degree relative was not included as a covariate in the multivariate model because the number of patients that reported family history of breast cancer in a first-degree relative was relatively small and rendered unstable estimates when included. We tested models excluding patients with a family history of breast cancer, and results were similar to those using the full dataset (**Table 6**).

**Table 6:** Multivariate multinomial logistic regression models testing the association between demographic and lifestyle variables and breast cancer subtype (HR+HER2− as reference).

		All patients*			Patients without FamHist		
Subtype	Variable	OR	95% CI	*p*-value	OR	95% CI	*p*-value
HR+HER2+	**Indigenous American ancestry** (Every 25% increment)	1.09	0.89–1.34	0.402	1.14	0.91–1.41	0.255
	**Age at diagnosis** (Every 5-year increment)	0.99	0.98–1.00	0.062	0.99	0.98–1.00	0.188
	**Height** (Every 1-cm increment)	1.01	0.99–1.03	0.257	1.01	0.99–1.04	0.196
	**Smoking history** (Ever *vs.* never [reference])	0.81	0.60–1.10	0.178	0.84	0.61–1.15	0.267
	**Parous** (Reference: nulliparous)	1.20	0.83–1.74	0.335	1.43	0.96–2.14	0.082
HR−HER2+	**Indigenous American ancestry** (Every 25% increment)	1.38	1.06–1.79	0.017	1.37	1.05–1.80	0.022
	**Age at diagnosis** (Every 5-year increment)	0.99	0.98–1.01	0.455	1.00	0.98–1.01	0.717
	**Height** (Every 1-cm increment)	0.98	0.96–1.01	0.166	0.99	0.96–1.01	0.266
	**Smoking history** (Ever *vs.* never [reference])	0.75	0.52–1.08	0.122	0.74	0.51–1.08	0.118
	**Parous** (Reference: nulliparous)	2.72	1.53–4.83	< 0.001	2.60	1.46–4.64	0.001
HR−HER2−	**Indigenous American ancestry** (Every 25% increment)	1.17	0.93–1.46	0.177	1.25	0.99–1.59	0.065
	**Age at diagnosis** (Every 5-year increment)	0.99	0.98–1.00	0.100	0.99	0.98–1.01	0.467
	**Height** (Every 1-cm increment)	1.01	0.99–1.03	0.446	1.02	0.99–1.04	0.205
	**Smoking history** (Ever *vs.* never [reference])	0.78	0.56–1.08	0.133	0.72	0.51–1.01	0.061
	**Parous** (Reference: nulliparous)	2.47	1.51–4.04	< 0.001	2.40	1.44–3.99	0.001

*Only samples for which genetic ancestry was available (n = 1,796) were included in this analysis. FamHist, family history of breast cancer in a first-degree relative (n = 1,628).

Indigenous American ancestry, region of residence, height, BMI, breastfeeding history, number of full-term pregnancies, and family history of breast cancer in a first-degree relative showed statistically significant associations at the 10% level with age at diagnosis categories. These variables were included in a multivariate model using age at diagnosis < 50 as reference (**Table 7**). We found that increasing Indigenous American ancestry and increasing height were associated with reduced odds of being diagnosed at 50 years or older (OR = 0.63, 95% CI = 0.53–0.75, *p* < 0.001 and OR = 0.96, 95% CI = 0.95–0.98, *p* < 0.001, respectively). Patients that resided in the Mountainous region had reduced odds of being diagnosed at 50 years of age or older compared with those in the Coastal region (OR = 0.63, 95% CI = 056–0.9, *p* = 0.004). Breastfeeding was associated with lower odds of being diagnosed at 50 years of age or older (OR = 0.35, 95% CI = 0.2–0.7, *p* = 0.001). Compared with nulliparous women, giving birth to at least one child increased the odds of being diagnosed at an older age (OR = 1.55, 95% CI = 0.2–0.7, *p* < 0.001). Increasing BMI was no longer associated with age at diagnosis (**Table 7**).

**Table 7:** Multivariate logistic regression model testing the association between demographic and lifestyle variables and age at diagnosis (< 50 [reference] *vs.* >= 50).

Variable	OR*	95% CI	*p*-value
**Indigenous American ancestry** (Every 25% increment)	0.63	0.53–0.75	< 0.001
**Height** (Every 1-cm increment)	0.96	0.95–0.98	< 0.001
**Region of residence** (Reference: Coastal region) Amazonian region Mountainous region	0.68 0.63	0.43–1.07 0.46–0.86	0.100 0.004
**BMI** (Every 1 kg/m2 increment)	1.02	1–1.05	0.080
**Parity** (Per each additional child)	1.55	1.43–1.69	< 0.001
**Breastfed** (Yes *vs*. no [reference])	0.35	0.20–0.70	0.001
Family history of breast cancer** (Yes *vs*. no [reference])	1.20	0.78–1.84	0.410

Only samples for which genetic ancestry was available (909 patients < 50 and 881 >= 50 years) were included in this analysis. *Patients diagnosed < 50 years old (reference) vs. >= 50 years. **In a first-degree relative.

## Discussion

In the present report, we aimed to provide a more complete description of the distribution of anthropometric, demographic, clinical, and known breast cancer–associated risk factors among Peruvian women that are part of The Peruvian Genetics and Genomics of Breast Cancer Study (PEGEN-BC). This work constitutes an update of a previously reported study, including a larger number of recruited patients and extending analyses to describe the distribution of patient characteristics not only by tumor subtype but also by age at diagnosis (62).

Being a hospital-based cohort, the PEGEN-BC study included a large proportion of women who resided in the Coastal region, where the INEN main hospital is located (**Figure 1**). Despite this bias in terms of residential representation, when looking at place of birth, the proportion of the cohort’s patients from the Coastal region followed closely that of the Peruvian population (58.0% Peru *vs.* 55.5% of cohort patients). The study has an overrepresentation of patients born in the Mountainous region (28.1% Peru *vs.* 36.4% of cohort patients) (68) and an underrepresentation of patients born in the Amazonian region (13.9% Peru *vs.* 7.5% of cohort patients) (68). The proportion of patients within each geographical region is consistent with what has been reported in two studies describing mortality of breast cancer (69) and incidence of triple-negative breast cancer tumors in Peruvian women (70).

A large proportion of patients were overweight/obese (67%), and the prevalence of exposure to alcohol and tobacco was higher than what has been previously reported for Peruvian women (71, 72). The average Indigenous American ancestry among the PEGEN-BC patients is 76.5%, which is higher than the average ancestry proportion of women in other breast cancer studies, including Latin America and U.S. Latinas (12, 51, 60, 73–89). In addition, the average height in our cohort was consistent with what has been reported in the literature for the Peruvian population (90) and with the known inverse correlation with Indigenous American ancestry (91). Overall, some reproductive variables showed a similar trend to what has been reported, including a similar age at menarche (92) and a high breastfeeding rate (93). The number of full-term pregnancies reported here (average of 2.8 children) was more closely related to what has been observed in rural areas of Peru (2.5) compared with urban areas (1.4) (94).

The distribution of tumor subtypes is similar to what has been previously described in other Latin American countries (95), with differences being partially explained by the inclusion of KI-67 expression and tumor grade for subtype classification (95), as indicated by the 2013 St. Gallen consensus (96). This classification criterion was not used in this report since KI-67 was not available for more than 20% of patients, and parameters for subtype determination based on this marker tend to be unstable across populations and studies (97). A study describing patient and tumor characteristics from Peruvian breast cancer patients at INEN diagnosed between 2000 and 2002 (80) (PEGEN-BC patients were recruited if diagnosed in 2010 or later) reported a lower proportion of HR+ tumors compared with PEGEN-BC (62.5% *vs.* 71.1%). This difference is likely to be explained by the higher positivity percentage cutoff value for HR+ status used in the previous report (10%, compared with 1% in PEGEN-BC), increasing the proportion of HR+ tumors in our cohort. Other characteristics, such as age at diagnosis and stage, presented similar distribution to the PEGEN-BC study cohort.

We found statistically significant differences by tumor subtype for Indigenous American genetic ancestry and height. In addition, we observed suggestive associations for age at diagnosis, family history of breast cancer in a first-degree relative and tobacco exposure. Differences were mostly driven by the HR−HER2+ subtype. Among patients with HR−HER2+ disease, we observed that the average height was lower compared with patients diagnosed with other tumor subtypes and was less likely to report smoking or a positive family history of breast cancer in a first-degree relative. Even though subtype-specific associations have been reported for these variables in other populations (38, 98–101), results in the Peruvian cohort showed that of all the above variables Indigenous American ancestry proportion was the only one that was differentially distributed by tumor subtype in multivariable models.

We did not find statistically significant differences for age at menarche by tumor subtype. Some studies have shown consistent associations between age at menarche and reduced risk of HR+HER2− breast cancer (3, 19, 20). One multicenter study did not find subtype-specific associations (27), consistent with our study. The PRECAMA Study, a Latin American population-based case-control study of premenopausal breast cancer, reported reduced odds for HR− tumors among women who were > 12 years old at menarche, compared with those younger at menarche (26, 51). In the current study, we did not find a statistically significant difference in average age at menarche by tumor subtype despite the observed correlation between the former and Indigenous American ancestry proportion.

We observed a higher frequency of parous women diagnosed with HR− subtypes compared with HR+. Parity (ever *vs.* never) has been associated with a higher risk of HR−HER2− subtypes, especially among women of African origin (33–35). Higher number of full-term pregnancies has been associated with reduced breast cancer risk (19, 31), with lower odds of developing HR+ tumors (3, 19, 20, 24–27, 29–35). We found significant differences in number of births by subtype, being higher among HR− subtypes compared with HR+ (2.7 compared with 2.3, respectively). Results suggested a larger proportion of women with > 3 children among those with HR− disease subtypes. This observation was consistent with studies in African American women reporting a higher number of reported full-term pregnancies among women with HR− disease (33). Studies that have tested the association between age at first full-term pregnancy and subtype-specific risk have shown a decreased risk of developing HR+HER2− tumors with unclear associations for other subtypes (25, 27, 31). In African American cohorts, limited breastfeeding among parous women is associated with an increased risk for HR−HER2− subtypes (34). The current study does not include detailed pregnancy and lactation history for the patients. As a result, we could not assess the association between time to breastfeeding cessation and cumulative time of breastfeeding and HR− subtypes.

There were statistically significant differences in the prevalence of demographic, anthropometric, and reproductive factors by age at diagnosis categories. The multivariate analysis showed that these variables are independently associated with age at diagnosis. Moreover, the differences in BMI by age at diagnosis were concordant with what is known about pre- and post-menopausal–specific disease risk factors (39–43). It must be considered that the observed differences in parity and height by age at diagnosis could be due to the correlation between age and the former (i.e., number of children and height are positively correlated with age) and not to an association between those variables and pre- *versus* post-menopausal disease.

The observed association between tumor subtype and Indigenous American ancestry could be due to a multiplicity of factors that we might not have collected information on in the PEGEN-BC study. For example, the study did not obtain information on the level of education or socioeconomic status of participants; both variables were previously shown to be associated with Indigenous American ancestry) among U.S. Latinas and Mexican women (76, 102, 103). Socioeconomic status can also impact screening, which in turn can affect tumor subtype distribution and mortality rates. Reports showed that less than 20% of Peruvian women 40–59 years of age have had a mammography, with vast differences according to socioeconomic status, educational level, health insurance, and region of residence (104, 105). Plan Esperanza, launched in 2012, has aimed to provide universal cancer screening and decentralize oncological health care across Peru, focusing on underserved commuties (106).

The PEGEN-BC study had some additional limitations. First, since menopause can be induced by treatment, most of the PEGEN-BC participants were postmenopausal at the time of the interview (86%). Therefore, we did not perform stratification by menopausal status and used age at diagnosis (< 50 *vs.* >= 50) instead to differentiate early onset *versus* late onset disease, as it has been widely used in epidemiological studies (107, 108). Even though menopausal status and age at diagnosis are highly correlated, studies have shown that age at diagnosis is a driver for breast cancer heterogeneity, acting as a confounder in analyses stratified by menopausal status (109). For this reason, the use of age as a proxy for menopausal status should be taken with caution. The second limitation concerns the relatively low variability of some of the assessed factors among PEGEN-BC study participants. For example, the assessment of the association between breastfeeding and the number of births and tumor subtype was hampered by the low prevalence of women without children and of women with children who did not breastfeed them. Additionally, we described the distribution of multiple factors across tumor subtypes, which provide evidence of heterogeneity; however, future case-control design studies should further explore subtype-specific breast cancer risk. Finally, average East Asian and African genetic ancestry components showed differences by subtypes in the univariate analyses. However, since ancestry estimates are correlated, and the proportions of East Asian and African genetic ancestries were relatively low as to provide reliable estimates, we focused the current description on the Indigenous American ancestry, which is the dominant component in Peruvians.

In summary, results confirmed the previously reported higher average Indigenous American ancestry among patients with HR−HER2+ breast cancer in this larger sample of PEGEN-BC study participants. Moreover, differences in tumor subtype by age at diagnosis were apparent and concordant with what is known about pre- and post-menopausal–specific disease associated risk factors. Larger studies are needed to understand the consistently observed association between ancestry, age of onset, and disease subtypes, considering the contribution of screening and treatment, to develop population-appropriate predictive models and targeted outreach and prevention campaigns.

## Data availability statement

All data supporting the conclusions of this article will be made available by the authors, without undue reservation.

## Ethics statement

The studies involving human participants were reviewed and approved by University of California Davis Institutional Review Boards and the Instituto Nacional de Enfermedades Neoplásicas (INEN). The patients/participants provided their written informed consent to participate in this study. Written informed consent was obtained from the individual(s) for the publication of any potentially identifiable images or data included in this article.

## Author contributions

LF= Conceptualization, Methodology, Investigation, Formal Analysis, Writing- Review and editing, Supervision, Project administration, and Funding acquisition. VZ: Methodology, Investigation, Formal Analysis, Writing- Original Draft, Software, Data curation, and Visualization. TV= Conceptualization, Resources, Project administration at INEN. SC-Z= Resources, Project administration at INEN. JN-V= Investigation, Data curation. CC, GV, MC, JA, HG, HF, RL-P, JC, SN, KR, JV, LM, MG-N= Conducted patient recruitment investigation process. All authors contributed to the article and approved the submitted version.
