# Supplementary material for: Breast cancer subtype and clinical characteristics in women from Peru
Source: Front Oncol. 2023 Feb 16;13:938042. doi: 10.3389/fonc.2023.938042 (PMC10013058; doi:10.3389/fonc.2023.938042)
Supplement: Supplementary file 1 [file Table_1.docx]

Supplementary Material

**Supplementary table S1**: Distribution of demographic and anthropometric variables by hormone receptor status.

|  | **Hormone Receptor (HR) Status** | |  |
| --- | --- | --- | --- |
| **Variable** | **HR+** | **HR-** | **p-value** |
| Number of patients, N (%) | 1282 | 520 |  |
| **Demographic variables** | | | |
| Age at diagnosis in years, mean (SD) | 49.95 (10.86) | 49.34 (11.49) | 0.293 |
| Percent genetic ancestry*, mean (SD) |  |  |  |
| Indigenous American | 0.76 (0.17) | 0.78 (0.16) | 0.002 |
| European | 0.18 (0.13) | 0.17 (0.12) | 0.014 |
| African | 0.04 (0.08) | 0.04 (0.07) | 0.452 |
| East Asian | 0.02 (0.08) | 0.01 (0.03) | 0.023 |
| Region of birth, N (%) |  |  |  |
| Amazonian | 91 (7.1) | 41 (7.9) | 0.691 |
| Coastal | 700 (54.6) | 289 (55.6) |  |
| Mountains | 483 (37.7) | 186 (35.8) |  |
| Other Country** | 8 (0.6) | 4 (0.8) |  |
| Region of Residence, N (%) |  |  |  |
| Amazonian | 74 (5.8) | 35 (6.7) | 0.091 |
| Coastal | 1021 (79.6) | 390 (75.0) |  |
| Mountains | 187 (14.6) | 95 (18.3) |  |
| **Anthropometric and lifestyle variables** | | | |
| Weight in kg, mean (SD) | 65.07 (12.24) | 64.14 (12.21) | 0.151 |
| Height in cm, mean (SD) | 153.43 (6.51) | 152.83 (6.51) | 0.081 |
| BMI in kg/m^2^, mean (SD) | 27.60 (4.76) | 27.45 (4.86) | 0.553 |
| BMI categorized, N (%) |  |  |  |
| Underweight** | 15 (1.2) | 6 (1.2) | 0.978 |
| Normal | 372 (29.0) | 149 (28.7) |  |
| Overweight | 512 (39.9) | 210 (40.4) |  |
| Obese | 354 (27.6) | 137 (26.3) |  |
| Alcohol intake, N (%) |  |  |  |
| <1 glass/day | 878 (69.1) | 345 (66.9) | 0.467 |
| >1 glass/day | 91 (7.2) | 45 (8.7) |  |
| Never | 301 (23.7) | 126 (24.4) |  |
| Smoking history, N (%) |  |  |  |
| Never | 897 (70.0) | 391 (75.2) | 0.031 |
| Ever | 374 (29.2) | 125 (24.0) |  |

*Estimates of individual continental ancestry were available for 1796 (92%) patients. **Category not included in the Chi-square test due to small sample size

**Supplementary Table S2**: Distribution of reproductive and clinical variables by hormone receptor status.

|  | **Hormone Receptor (HR) Status** | |  |
| --- | --- | --- | --- |
| **Variable** | **HR+** | **HR-** | **p-value** |
| Number of patients, N (%) | 1282 | 520 |  |
| **Reproductive variables** | | | |
| Age at menarche in years, mean (SD) | 12.9 (1.75) | 13.0 (1.66) | 0.182 |
| Age at first full-term pregnancy in years, mean (SD) | 23.4 (5.64) | 22.7 (5.74) | 0.043 |
| Parous, yes, N (%) | 1038 (83.7) | 470 (92.7) | <0.001 |
| Parity, mean (SD) | 213 (17.1) | 41 (8.0) | <0.001 |
| Parity categories, N (%) |  |  |  |
| No child | 209 (16.8) | 84 (16.5) | <0.001 |
| 1 child | 571 (45.8) | 253 (49.6) |  |
| 2 to 3 children | 253 (20.3) | 132 (25.9) |  |
| >3 children | 209 (16.8) | 84 (16.5) |  |
| Breastfed*, yes, N (%) | 1000 (96.4) | 455 (96.8) | 0.827 |
| Postmenopausal, N (%) | 1126 (88.7) | 438 (85.2) | 0.049 |
| **Clinical characteristics** | | | |
| Positive family history of breast cancer**, N (%) | 109 (8.7) | 32 (6.3) | 0.111 |
| Grade, N (%) |  |  |  |
| 1 | 64 (5.1) | 4 (0.8) | <0.001 |
| 2 | 667 (53.1) | 73 (14.4) |  |
| 3 | 526 (41.8) | 431 (84.8) |  |
| Positive lymph node status, N (%) | 812 (66.0) | 353 (71.7) | 0.025 |
| Stage, N (%) |  |  |  |
| I | 85 (6.8) | 30 (5.8) | <0.001 |
| II | 614 (48.8) | 179 (34.9) |  |
| III | 490 (39.0) | 276 (53.8) |  |
| IV | 68 (5.4) | 1. 5.5) |  |

*Among parous women ** In a first-degree relative

**Supplementary table S3:** Distribution of demographic and anthropometric variables by tumor subtype and by age at diagnosis categories.

|  | Age at diagnosis <50 years* | | | | | Age at diagnosis >=50 years** | | | | |
| --- | --- | --- | --- | --- | --- | --- | --- | --- | --- | --- |
| **Variable** | HR+HER2- | HR+HER2+ | HR-HER2+ | HR-HER2- | p-value | HR+HER2- | HR+HER2+ | HR-HER2+ | HR-HER2- | p-value |
| Number of patients, N (%) | 476 (48.5) | 178 (18.1) | 108 (11.0) | 155 (15.8) |  | 468 (49.0) | 159 (16.6) | 124 (12.3) | 132 (13.8) |  |
| **Demographic variables** | | | | | | | | | | |
| Age at diagnosis in years, mean (SD) | 41.4 (5.6) | 41.1 (5.5) | 40.8 (6.5) | 39.9 (6.7) | 0.063 | 59.4 (7.2) | 57.7 (6.3) | 57.9 (7.0) | 59.3 (7.5) | 0.023 |
| Percent genetic ancestry, mean (SD) |  |  |  |  |  |  |  |  |  |  |
| Indigenous American | 78.5 (15.1) | 78.5 (14.0) | 78.6 (15.7) | 78.4 (16.5) | 1.000 | 72.2 (19.0) | 74.5 (19.2) | 80.2 (13.9) | 76.6 (16.6) | <0.001 |
| European | 17.0 (11.5) | 16.6 (10.4) | 16.9 (11.8) | 16.9 (12.5) | 0.989 | 20.5 (13.9) | 19.1 (13.5) | 15.5 (10.9) | 17.9 (12.7) | 0.003 |
| African | 3.3 (6.3) | 4.1 (6.2) | 3.8 (6.4) | 4.0 (8.0) | 0.388 | 4.87 (8.6) | 5.2 (11.2) | 3.4 (6.2) | 4.3 (6.1) | 0.321 |
| East Asian | 1.3 (5.7) | 0.8 (1.9) | 0.7 (1.7) | 0.7 (1.6) | 0.314 | 2.51 (10.7) | 1.3 (5.0) | 0.8 (2.9) | 1.2 (4.2) | 0.112 |
| Region of birth, N (%) |  |  |  |  |  |  |  |  |  |  |
| Amazonian | 36 (7.6) | 11 (6.2) | 8 (7.4) | 11 (7.1) | 0.414 | 33 (7.1) | 11 (6.9) | 10 (8.1) | 12 (9.1) | 0.728 |
| Coastal | 255 (53.6) | 91 (51.1) | 64 (59.3) | 94 (60.6) |  | 266 (56.8) | 87 (54.7) | 60 (48.4) | 70 (53.0) |  |
| Mountains | 181 (38.0) | 76 (42.7) | 35 (32.4) | 48 (31.0) |  | 165 (35.3) | 61 (38.4) | 53 (42.7) | 50 (37.9) |  |
| Other Country*** | 4 (0.8) | 0 (0.0) | 1 (0.9) | 2 (1.3) |  | 4 (0.9) | 0 (0.0) | 1 (0.8) | 0 (0.0) |  |
| Region of Residence, N (%) |  |  |  |  |  |  |  |  |  |  |
| Amazonian | 30 (6.3) | 10 (5.6) | 5 (4.6) | 14 (9.0) | 0.622 | 26 (5.6) | 8 (5.0) | 6 (4.8) | 10 (7.6) | 0.053 |
| Coastal | 365 (76.7) | 130 (73.0) | 82 (75.9) | 116 (74.8) |  | 391 (83.5) | 134 (84.3) | 92 (74.2) | 100 (75.8) |  |
| Mountains | 81 (17.0) | 38 (21.3) | 21 (19.4) | 25 (16.1) |  | 51 (10.9) | 17 (10.7) | 26 (21.0) | 22 (16.7) |  |
| **Anthropometric and lifestyle variables** | | | | | | | | | | |
| Weight in kg, mean (SD) | 65.10 (12.0) | 65.01 (11.8) | 63.03 (11.1) | 64.91 (13.5) | 0.449 | 65.24 (12.8) | 64.52 (12.1) | 64.02 (12.0) | 64.19 (11.7) | 0.698 |
| Height in m, mean (SD) | 154.06 (6.4) | 154.03 (5.9) | 153.18 (6.5) | 154.46 (6.4) | 0.444 | 152.54 (6.6) | 153.40 (6.9) | 151.13 (6.4) | 152.08 (6.4) | 0.041 |
| BMI in kg/m^2^, mean (SD) | 27.41 (4.7) | 27.31 (4.6) | 26.84 (4.4) | 27.13 (4.9) | 0.690 | 27.98 (4.9) | 27.44 (5.0) | 28.07 (5.3) | 27.77 (4.8) | 0.648 |
| BMI categorized, N (%) |  |  |  |  |  |  |  |  |  |  |
| Underweight*** | 2 (0.4) | 1 (0.6) | 1 (0.9) | 2 (1.3) | 0.414 | 9 (2.0) | 3 (1.9) | 3 (2.6) | 0 (0.0) | 0.314 |
| Normal | 144 (30.9) | 59 (33.9) | 30 (28.0) | 50 (33.3) |  | 118 (25.9) | 50 (32.1) | 35 (30.4) | 34 (26.4) |  |
| Overweight | 200 (42.9) | 69 (39.7) | 55 (51.4) | 56 (37.3) |  | 183 (40.1) | 60 (38.5) | 38 (33.0) | 61 (47.3) |  |
| Obese | 120 (25.8) | 45 (25.9) | 21 (19.6) | 42 (28.0) |  | 146 (32.0) | 43 (27.6) | 39 (33.9) | 34 (26.4) |  |
| Alcohol intake, N (%) |  |  |  |  |  |  |  |  |  |  |
| <1 glass/day | 325 (68.3) | 118 (66.3) | 69 (63.9) | 100 (64.5) | 0.938 | 329 (71.2) | 105 (66.5) | 90 (72.6) | 85 (64.9) | 0.658 |
| >1 glass/day | 40 (8.4) | 14 (7.9) | 10 (9.3) | 15 (9.7) |  | 26 (5.6) | 11 (7.0) | 9 (7.3) | 11 (8.4) |  |
| Never | 106 (22.3) | 46 (25.8) | 26 (24.1) | 40 (25.8) |  | 107 (23.2) | 42 (26.6) | 25 (20.2) | 35 (26.7) |  |
| Smoking history, N (%) |  |  |  |  |  |  |  |  |  |  |
| Never | 337 (71.4) | 133 (74.7) | 81 (77.1) | 107 (69.0) | 0.429 | 318 (68.8) | 109 (69.0) | 98 (79.0) | 104 (79.4) | 0.023 |
| Ever | 135 (28.6) | 45 (25.3) | 24 (22.9) | 48 (31.0) |  | 144 (31.2) | 49 (31.0) | 26 (21.0) | 27 (20.6) |  |

*Immunohistochemical subtype classification was not available for 64 samples (6.5%). **Immunohistochemical subtype classification was not available for 72 samples (7.5%) *** Category not included in the Chi-square test due to small sample size

**Supplementary table S4**: Distribution of reproductive and clinical variables by subtype and by age at diagnosis categories.

|  | Age at diagnosis <50 years* | | | | | | Age at diagnosis >=50 years** | | | | |
| --- | --- | --- | --- | --- | --- | --- | --- | --- | --- | --- | --- |
| **Variable** | HR+HER2- | HR+HER2+ | HR-HER2+ | HR-HER2- | p-value | HR+HER2- | | HR+HER2+ | HR-HER2+ | HR-HER2- | p-value |
| Number of patients, N (%) | 476 (48.5) | 178 (18.1) | 108 (11.0) | 155 (15.8) |  | 468 (49.0) | | 159 (16.6) | 124 (12.3) | 132 (13.8) |  |
| **Reproductive variables** | | | | | | | | | | | |
| Age at menarche in years, mean (SD) | 12.88 (1.8) | 12.96 (1.6) | 13.10 (1.7) | 12.91 (1.7) | 0.680 | 12.94 (1.8) | | 12.86 (1.8) | 13.16 (1.6) | 12.98 (1.6) | 0.522 |
| Age at first full-term pregnancy in years, mean (SD) | 23.70 (5.7) | 23.01 (5.4) | 22.87 (5.7) | 22.40 (5.2) | 0.098 | 23.25 (5.8) | | 22.99 (5.2) | 22.98 (6.5) | 22.71 (5.8) | 0.779 |
| Parous, yes, N (%) | 380 (83.0) | 150 (87.2) | 96 (90.6) | 140 (91.5) | 0.024 | 384 (84.6) | | 123 (79.4) | 111 (94.9) | 122 (93.8) | <0.001 |
| Parity categories, N (%) |  |  |  |  |  |  | |  |  |  |  |
| 0 children*** | 84 (18.2) | 24 (13.9) | 11 (10.3) | 14 (9.1) | 0.069 | 72 (15.8) | | 33 (21.2) | 8 (6.8) | 8 (6.2) | 0.003 |
| 1 child | 103 (22.3) | 32 (18.5) | 18 (16.8) | 29 (18.8) |  | 59 (13.0) | | 15 (9.6) | 20 (16.9) | 17 (13.1) |  |
| 2 to 3 children | 225 (48.8) | 94 (54.3) | 61 (57.0) | 89 (57.8) |  | 184 (40.4) | | 67 (42.9) | 44 (37.3) | 58 (44.6) |  |
| >3 children | 49 (10.6) | 23 (13.3) | 17 (15.9) | 22 (14.3) |  | 140 (30.8) | | 41 (26.3) | 46 (39.0) | 47 (36.2) |  |
| Breastfed, yes, N (%) | 371 (97.6) | 149 (99.3) | 95 (99.0) | 137 (97.9) | 0.541 | 364 (95.0) | | 115 (93.5) | 105 (94.6) | 117 (95.9) | 0.854 |
| **Clinical characteristics** | | | | | | | | | | | |
| Positive family history of breast cancer****, N (%) | 28 (6.0) | 11 (6.3) | 3 (2.9) | 17 (11.0) | 0.053 | 56 (12.3) | | 14 (8.9) | 6 (5.0) | 6 (4.6) | 0.014 |
| Grade, N (%) |  |  |  |  |  |  | |  |  |  |  |
| 1 | 30 (6.5) | 3 (1.7) | 0 (0.0) | 2 (1.3) | <0.001 | 28 (6.1) | | 3 (1.9) | 0 (0.0) | 2 (1.6) | <0.001 |
| 2 | 268 (57.8) | 63 (35.8) | 17 (16.0) | 13 (8.6) |  | 282 (61.2) | | 54 (34.6) | 19 (15.6) | 24 (18.6) |  |
| 3 | 166 (35.8) | 110 (62.5) | 89 (84.0) | 136 (90.1) |  | 151 (32.8) | | 99 (63.5) | 103 (84.4) | 103 (79.8) |  |
| Positive lymph node status, N (%) | 302 (67.0) | 116 (66.7) | 87 (83.7) | 92 (63.9) | 0.004 | 282 (62.7) | | 111 (72.1) | 89 (73.6) | 85 (69.7) | 0.039 |
| Stage, N (%) |  |  |  |  |  |  | |  |  |  |  |
| I | 24 (5.2) | 9 (5.1) | 2 (1.9) | 8 (5.3) | 0.001 | 43 (9.3) | | 9 (5.8) | 5 (4.0) | 15 (11.5) | <0.001 |
| II | 237 (50.9) | 75 (42.9) | 29 (27.4) | 66 (43.7) |  | 242 (52.5) | | 59 (38.3) | 41 (33.1) | 42 (32.1) |  |
| III | 179 (38.4) | 83 (47.4) | 68 (64.2) | 70 (46.4) |  | 153 (33.2) | | 75 (48.7) | 69 (55.6) | 69 (52.7) |  |
| IV | 26 (5.6) | 8 (4.6) | 7 (6.6) | 7 (4.6) |  | 23 (5.0) | | 11 (7.1) | 9 (7.3) | 5 (3.8) |  |

*Immunohistochemical subtype classification was not available for 64 samples (6.5%) **Immunohistochemical subtype classification was not available for 72 samples (7.5%) *** Category not included in the Chi-square test due to small sample size **** In a first-degree relative
